# A Reviewer Reviewed

**Published:** 1889-06

**Authors:** 


BUFFALO MEDICAL AND SURGICAL JOURNAL
A MONTHLY REVIEW OF MEDICINE AND SURGERY.

EDITORS:
THOS. LOTHROP, M. D., - - - W. W. POTTER, M. D.

All communications, whether of a literary or business character, should be addressed to the editors: 284 FRANKLIN STREET, BUFFALO, N. Y.

### Editorial.

### *A REVIEWER REVIEWED.*[1]

NO MORE fitting point can be chosen for the renewal of our comment upon "J. C. R.'s" review of Mr. Tait's lectures than the apparently studied misrepresentation of Mr. Tait's views of the much-discussed subject of ovarian pregnancy.

"J. C. R." would have the reader believe that Spiegelberg's authority is set at naught, while the truth is, the greatest deference is paid the German author, as the following attests:

"I frankly admit that the eminence of the observer, and the manifest care with which all his records are given, make it quite possible that his conclusions are correct" [p. 11].

So in Puech's case, Mr. Tait's attitude of skepticism is twisted and distorted by this amazing, critical acrobat, until his doubt becomes denial. Mr. Tait says:

"Of course, the whole conclusion in this case depends upon the assumption that this vermiform body, ONLY ONE MM. LONG, was an embryo. It may have been one, but certainly there is no proof advanced in favor of this view; *and although I am by no means prepared to deny its accuracy, I am certainly very doubtful about it. . . . . . I have seen so many queer-looking things in ovarian cysts and follicles*, that I am not inclined to admit that this vermiform body has been shown conclusively to have been an embryo" [p. 11].

The portion we have italicized is omitted altogether by 'J. C. R." One would expect such evasion and omission and

1. Continued from May number, page 614.

verbal juggling in barristers' quarrels or practical politics, certainly not in a scientific review.

The fact that Mr. Tait does not refer to Spiegelberg's assertion of ten proven cases, nor to Leopold's of fourteen, is evidenced as dereliction. This attack evidently taking its origin in a desire to criticize, culminates in the disbelief that the subject-matter of the book should be considered as "Lectures." Had the reviewer considered for a moment that the author was not writing an exhaustive treatise, but simply "lecturing," he would have understood that it was amply sufficient to cite one or two typical cases, accurately reported, and to weigh them from his personal standpoint, and so far take them as a measure of the value of other cases. Tait's citation of the authority of Willigh disproving the genuineness of many reported cases of ovarian pregnancy, though due reference is given, is passed over without remark, even though the case of Kiwisch is thereby deprived of a place in this list. There is little use of further comment upon the subject, as reviewed by "J. C. R." When we consider that the author of the lectures unequivocally admits the possibility of this variety of ectopic gestation, though questioning its probability, the utter unreliability of "J. C. R.'s" critical judgment and statement in his review, is at once rendered apparent. Says Tait:

"Parry sums up the subject by saying that the weight ot authority is in favor of the possibility of ovarian pregnancy. Its possibility I admit because I can easily imagine a Fallopian tube glued on to the ovary, and deprived of its lining epithelium, permitting the contact of the spermatozoa, with a follicle burst within the area [of the ovary] of adhesion. Then the spermatozoa might infect the ovum before it escaped from the follicle, the ovum might adhere to the follicular wall and then develop. But there are so many contingencies in such a case, that the doctrine of chance makes it so remote that its occurrence may be regarded as likely as the birth of a blue lion, or a swan with two necks, like a heraldic monstrosity, a mere pathological curiosity. If it does occur, it most be rare and will be curious. If it never occurs, so much the better" [p. 13].

The strange part of all this is, that the author's right to demand exact proof of conditions he has not seen, is denied, and this in spite of this enormous experience. When "J. C. R." says, "The ovarian variety of extra-uterine pregnancy is, undoubtedly, very rare," [p. 375,] it will be seen he agrees exactly with what has been quoted above. Such being the case, why pretend, distort, exaggerate, misquote, evade, and even approach the limits of absolute falsification? Is it simply to appear learned, by the sacrifice of honesty?

The next point worthy of attention, is the question of the occurrence of primary abdominal pregnancy. Mr. Tait does not believe it probable,— possible, if we choose,— for this accident to take place. He says:

"That a fertilised ovum may drop into the cavity of the peritoneum and become developed there, is a contingency I cannot accept for a moment, for the powers of digestion of the peritoneum are so extraordinary, that an ovum, even if fertilised, could have no chance of development."

"J. C. R." stops here, but Mr. Tait continues:

"What have been called abdominal pregnancies, are clearly exceptional cases where primary tubal rupture at the end of the third month has not proved fatal, where the extruded placenta has made for itself visceral attachments wherever it has touched, or where secondary rupture of broad ligament cyst has converted an extra-peritoneal ectopic gestation into one within the peritoneal cavity" [p. 13].

Here are two explanations of a pathological condition which are passed over as if worthless. We believe, however, that to the average surgical intelligence, outside the field of paid criticism, or private pique, they will appear as strongly probable, and much more satisfactory than any other.

Says "J. C. R.": "There have been some cases in which the placenta was found attached to omentum and intestines" [p. 375]. Says Mr. Tait: "Similarly I have seen this strange transplantation in all its phases and in all stages of the process" [p. 15]. This, Mr. Tait believes, explains Lecluyse's and Maticki's cases. And why not? Does "J. C. R.'s" citation of Kellar's case prove anything beyond this, that the unexpected always happens? It would ordinarily be very difficult to imagine the occurrence of pregnancy in a woman whom both the body and a portion of the cervix uteri had been amputated. It is certainly no harder to believe such pregnancy tubal, than to conceive the possibility of the development of a microscopic ovum in the peritoneal fluids, especially as no proof is adduced to fortify this opinion.

"J. C. R.'s" citation of Thomas's case is still further proof of his disingenuity. Thomas reports this case in *Trans. Am. Gyn. Soc.*, Vol. IX., p. 178. He says:

"The patient passed through a period of gestation, 'marked by the occurrence of abdominal pain, occasional hemorrhage, tympanitis fever, etc.'"

Is anything plainer than that this case was one originally tubal, finally rupturing into the abdominal (peritoneal) cavity? Thomas himself does not even suggest the possibility of the case being an unusual variety of ectopic pregnancy. This being so, why should "J. C. R." appropriate it, and label it as such? The audacity of his performance is amazing.

We come now to the consideration of the diagnosis and treatment of ectopic gestation previous to rupture. Here for the first time "J. C. R." has many facts from reliable witnesses to bear out what he says concerning these mooted questions. The pity is that, with so much reason and authority on his side, even here he is as untrustworthy as ever, by reason of misstatements, and of making half-truths stand duty for facts. Quoting from Bernutz and Goupil, he speaks of an "ensemble of signs, no one of which is pathognomonic, but the reunion of which scarcely leaves a doubt" as to the existence of extra-uterine pregnancy. Why does he not continue the quotation, when a few more lines would have discovered the following?

"Undoubtedly the concurrence of these two sets of symptoms should at least make us *suspect* the existence of extra-uterine pregnancy, and this *suspicion* will grow into a *certainty* when the complications of rupture, and internal hemorrhage supervene. I attach great importance to the phenomena which precede these hemorrhages, and which alone help us to a correct diagnosis." (Bernutz and Goupil, Vol. I., page 265, Syd. Soc.)

Mr. Tait insists upon the essential guess-work of the diagnosis of extra-uterine pregnancy. After rupture, he believes diagnosis can be made seven times out of eight, and guessed at the eighth.

To say that the condition has never been diagnosticated previous to rupture, would be asserting too much. To say that such diágnosis is as often wrong as correct, is probably also saying too much for it. Herein lies the weakness of most of the diagnoses in patients to whom electricity is afterwards applied.

Buckmaster's case, so strongly condemned by Mr. Tait, and championed by "J. C. R.," *Medical News*, July 21, 1888, granting for the sake of argument that the diagnosis was correct, is entirely offset by another of Buckmaster's own cases, in which electricity was applied to kill the fetus of an extra-uterine pregnancy unavailingly, and finally the woman is set free to have her labor by what, it is hoped, will be yet regarded for a long time as the natural way. This case has never been published, so far as we know.

In "J. C. R.'s" review, *American Journal of the Medical Sciences*, page 377, are two references to the published discussions upon the diagnosis of extra-uterine pregnancy. It is almost past belief that both of these are garbled and made to appear as condemning, at the present time, their author, Mr. Tait, by his own previous assertions. However, such, alas! is the case. The first reference, *American Journal of the Medical Sciences*, July, 1873, page 278, reports Mr. Tait as saying (also in his treatise on Diseases of the Ovaries, page 83,):

"That in extra-uterine pregnancy there is a tumor intimately associated with the uterus, which is always enlarged, and that a cervix quite open is the most important point."

"J. C. R." here comments: "In this, his latest production, Mr. Tait is behind himself." Let us see. We quote more at length Mr. Tait's discussion:

"The importance had been overlooked in this case of the absence of retro-uterine fulness, or rather the absence of a solid tumor there. It would bé almost impossible to imagine a case of extra-uterine fetation without a retro-uterine tumor, giving to the finger a feeling of cystic ballottement, previous to the absorptions of the amniotic fluid, but after that feeling solid. . . . There were two circumstances which invariably accompanied extra-uterine gestation which has gone past the period. . . . After the death of the child the diagnosis is more difficult. . . . . . . The uterus in extra-uterine pregnancy is always associated with a tumor, and generally in front of it, movable to a certain extent, and enlarged. The most important point was that the cervix is always patulous. Under such circumstances, if a fetal heart were audible, the case was clear."

Now, when it is understood that this discussion was made after the reports of three cases of extra-uterine pregnancy, in one of which an error was made, there being no pregnancy at all; and, in another, the woman had missed labor; while, in the third case, no diagnosis was made until the fetal heart was heard, the fallacy of making Mr. Tait testify to the possibility of absolute and positive diagnosis previous to rupture in this discussion, is apparent. We do not agree, be it understood, that there are no symptoms of extra-uterine gestation; neither, we submit, does Mr. Tait mean to say there are none. To have made himself plain past carping, he should have said, as is his obvious meaning, that there are no *uniform* symptoms, and none which are *infallible*. Unfortunately again, for the reviewer, almost this entire discussion is found in the Lectures, p. 67, so that, after all, his duplicity is again betrayed, and Mr. Tait is not "behind himself."

The reference to the *British Medical Journal*, June 28, 1884, p. 1250, reports Mr. Tait as saying: "The diagnosis is not so very difficult after all." Here, as before, our critic pauses, as if suddenly afflicted with blindness. He should have read on briefly, when he would have met the following:

"It may be in the majority, however, that this misleading feature is present if the patient has never been pregnant, or has not been so for many years, and then the arrest of menstruation attracts no particular attention. If, however, it be found that the patient has been eight weeks or more without a period, that there is a pelvic mass fixing the uterus and on one side of it, and that sudden and severe symptoms of pelvic trouble and hemorrhage come on, the rupture of a tubal pregnancy may be at once suspected."

Here again it is evident that the writer's testimony is as to his belief in the possibility of a diagnosis after rupture. This he insists upon in his Lectures. "J. C. R.'s" misrepresentation is wilful and shameless. We should expect such chicanery not in a scientific review but rather in a spiritualistic séance.

We now pass on to a brief consideration of the treatment of ectopic gestation. Here again "J. C. R." might have dealt calmly and judicially with a subject which, to say the least, is still *sub judice*. That Mr. Tait seems to have confounded the electrolytic puncture of fibroids with the simple passage of the faradic current through the ectopic mass, is evident, though, so far as his argument is concerned, this makes little difference. His position briefly stated is this: "The extra-uterine pregnancy is a foreign mass. If it is recognized before rupture, remove it." This doctrine, to which we heartily subscribe, has no place for electricity in any shape. Granted that electricity does all that is claimed for it; grant that all the so-called diagnoses are actually made,—the case stands the same. The fetus is only killed, and remains ever afterward a menace to the safety of the woman. The fact that the fetus when left to itself, in a majority of the cases, dies and suppurates through the abdominal walls or elsewhere, strongly sustains the position of Mr. Tait, that it is dangerous to kill it and leave it. The weak point in Mr. Tait's argument is, we think, his hesitating, for moral reasons, to operate when the child is found to have survived primary rupture. His position would be more defensible, were secondary rupture never fatal.

That the placenta grows after the death of the fetus, is so well attested by Champneys, Thornton, and Hart and Barbour, that "J. C. R.'s" skepticism can be allowed to stand. Whether the placenta will grow after feticide by electricity, is perhaps undecided. Mr. Tait's fear concerning this may be without foundation, yet the law that "an organ whose function has ceased atrophies," does not always apply to the placenta.

Space forbids a further examination of this "review." We have devoted time and attention to it at such a length for two purposes: first, to defend the position of the author reviewed, who we think is in the main correct in his opinions [noticed in this JOURNAL, March, 1889,]; and second, to rebuke and expose

"J. C. R.," his reviewer; for it is important that the views of an author, particularly upon a subject of such great importance to every general practitioner, shall not be distorted, warped, or overcolored by his critic. Honest healthy criticism is commendable; such as we have just arraigned is disreputable. It is rank and offensive. It betrays the confidence of the readers of the journal in which it appears, who desire for their enlightenment on such topics neither spleen nor fiction, but justice and truth. Any tendency "J. C. R." has toward the latter, he has diligently restrained.

Laying down the lines for a scientific discussion, he says:

"In an open debate upon any subject, it is held essential to honor that the position of opponents shall be fairly stated, and whatever facts they present be treated with justice."

"J. C. R." manifestly belongs to that class of "ungracious pastors" who

> "Show '*us*' the steep and thorny way to heaven,
> Whiles, like a puff'd and reckless libertine,
> Himself the primrose path of dalliance treads,
> And recks not his own rede."

